# From quarks and gluons to baryon form factors

**DOI:** 10.1016/j.ppnp.2011.12.024

**Published:** 2012-04

**Authors:** Gernot Eichmann

**Affiliations:** Institut für Theoretische Physik, Universität Giessen, 35392 Giessen, Germany

**Keywords:** Nucleon, Delta, Form factors, Dyson-Schwinger equations, Faddeev equations

## Abstract

I briefly summarize recent results for nucleon and Δ(1232) electromagnetic, axial and transition form factors in the Dyson–Schwinger approach. The calculation of the current diagrams from the quark–gluon level enables a transparent discussion of common features such as: the implications of dynamical chiral symmetry breaking and quark orbital angular momentum, the timelike structure of the form factors, and their interpretation in terms of missing pion-cloud effects.

## 1 Introduction

Form factors encode basic structure properties of hadrons that are accessible in experiments. Probing hadrons with electromagnetic, axial and pseudoscalar currents reveals their underlying dynamics in terms of quarks and gluons in Quantum Chromodynamics (QCD). While the nucleon’s axial structure is experimentally more difficult to access, an abundance of information has been collected for photon-induced processes that are described by NNγ elastic and NΔγ transition form factors. Precision measurements have stimulated the development of new tools to address questions related to quark orbital angular-momentum correlations in the perturbative domain, the transition between perturbative and non-perturbative regions, or pion-cloud rescattering effects in the chiral and low-momentum region. The associated chiral non-analyticities stemming from the nucleon’s ‘pion cloud’ have been frequently discussed when connecting results from lattice QCD, chiral effective field theories and quark models with experiments.

**Fig. 1:**
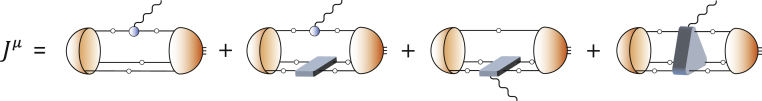
General expression for a baryon’s current matrix element in the Dyson–Schwinger/Faddeev approach. The $q\overset{¯}{q}$ vertex, dressed quark propagator, and qq and qqq kernels are sandwiched between incoming and outgoing baryon bound-state amplitudes. Only the first two terms survive in a rainbow-ladder truncation.

A complementary framework for studying hadron phenomenology is the one via Dyson–Schwinger equations (DSEs). They interrelate QCD’s Green functions and thereby provide access to nonperturbative phenomena such as dynamical chiral symmetry breaking and confinement, see [1], [2], [3] for reviews. Hadron properties are obtained from covariant bound-state equations, i.e., the Bethe–Salpeter equation for mesons and the covariant Faddeev equation for baryons. The respective current matrix elements are constructed by coupling an external current to all internal building blocks of the $q\overset{¯}{q}$ or qqq scattering matrices and taking their residues at the hadron bound-state poles. Fig. 1 illustrates the resulting form-factor diagrams for a baryon. They incorporate the qq and qqq interaction kernels which, once specified, allow for a self-consistent calculation of all further ingredients: the dressed quark propagator, the $q\overset{¯}{q}$ vertices that describe the current microscopically, and the nucleon and Δ bound-state amplitudes.

The investigation of hadron structure in the Dyson–Schwinger approach has several benefits. It is Poincaré-covariant throughout every step and provides access to all momentum scales and all quark masses without the need for extrapolations. Since one operates directly with QCD’s degrees of freedom, observable phenomena at the hadron level can be systematically traced back to their microscopic origin. Dynamical chiral symmetry breaking is realized and leads to a non-perturbative enhancement of the quark mass function and related propagators and vertices in QCD. If the kernels in Fig. 1 satisfy vector and axialvector Ward–Takahashi identities, electromagnetic current conservation at the hadron level, as well as the Gell–Mann–Oakes–Renner and Goldberger–Treiman relations, follow automatically. Moreover, the self-consistent calculation of the $q\overset{¯}{q}$ vertices that appear in Fig. 1 generates meson poles in the respective $J^{PC}$ channels which dictate the timelike structure of form factors.

The drawback of the approach is its necessity of truncations. The properties of baryons discussed herein have been obtained in a rainbow-ladder truncation, where qqq interactions are neglected and the qq and $q\overset{¯}{q}$ interaction is modeled by a dressed gluon exchange [4]. The Faddeev equation then generates all possible gluon ladder diagrams by iteration. The simplicity of that kernel entails that various phenomenologically important features are missed in the resulting form factors. A characteristic example is the absence of pion-cloud contributions in their chiral and low-momentum structure. The relevant gluon topologies that generate pion-cloud effects at the hadron level are not captured by a rainbow-ladder truncation which therefore represents the baryon’s ‘quark core’. In the case of the Δ form factors discussed below, an additional quark–diquark simplification is made, where scalar and axialvector diquark correlations approximate the qq scattering matrix and lead to an effective two-body description.

In the following we will summarize recent results for the nucleon’s electromagnetic, axial and NΔγ transition form factors. More detailed discussions, result tables as well as references for experimental and lattice data which are shown in the plots for comparison can be found in Refs. [5], [6], [7].

## 2 Nucleon electromagnetic form factors

The nucleon’s electromagnetic current is expressed by two dimensionless form factors: the Dirac and Pauli form factors $F_{1}\left(Q^{2}\right)$ and $F_{2}\left(Q^{2}\right)$, or the Sachs form factors $G_{E}\left(Q^{2}\right)$ and $G_{M}\left(Q^{2}\right)$ as their linear combinations: $G_{E}=F_{1}-Q^{2}/\left(4M_{N}^{2}\right)\;F_{2}$ and $G_{M}=F_{1}+F_{2}$. The current is given by

(1) $$J^{\mu }=i\Lambda_{+}\left(P_{f}\right)\left(F_{1}\left(Q^{2}\right)\;\gamma^{\mu }-F_{2}\left(Q^{2}\right)\;\frac{\sigma^{\mu \nu }Q^{\nu }}{2M_{N}}\right)\Lambda_{+}\left(P_{i}\right)\text{,}$$

where $Q=P_{f}-P_{i}$ is the photon momentum and $\Lambda_{+}\left(P\right)=\frac{1}{2}\;\left(1+P\;//\left(iM_{N}\right)\right)$ the nucleon’s positive-energy projector. In the static limit one retrieves the nucleons’ anomalous magnetic moments $\kappa =F_{2}\left(0\right)$ as well as their Dirac and Pauli radii $r_{1}^{2}=-6F_{1}^{\prime }\left(0\right)$ and $r_{2}^{2}=-6F_{2}^{\prime }\left(0\right)/F_{2}\left(0\right)$. The isoscalar (isovector) form factors are the sum (difference) of proton and neutron form factors: $F_{i}^{s}=F_{i}^{p}+F_{i}^{n}$ and $F_{i}^{v}=F_{i}^{p}-F_{i}^{n}$.

**Fig. 2:**
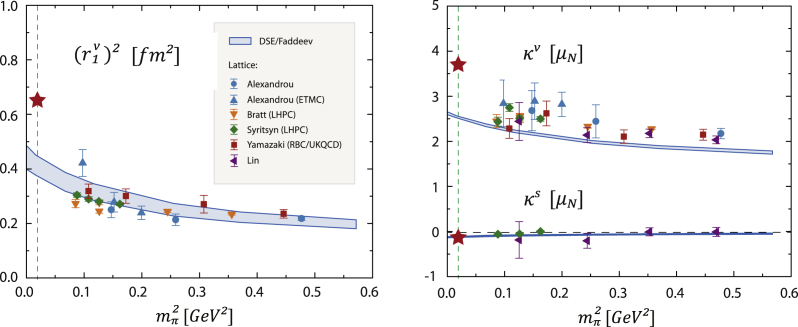
Quark-mass dependence of nucleon static electromagnetic properties compared to lattice results. *Left panel:* squared isovector Dirac radius ${\left(r_{1}^{v}\right)}^{2}$. *Right panel:* isovector and isoscalar anomalous magnetic moments $\kappa^{v}$ and $\kappa^{s}$ in units of nuclear magnetons. Stars denote the experimental values. Figure adapted from Ref. [5].

**Fig. 3:**
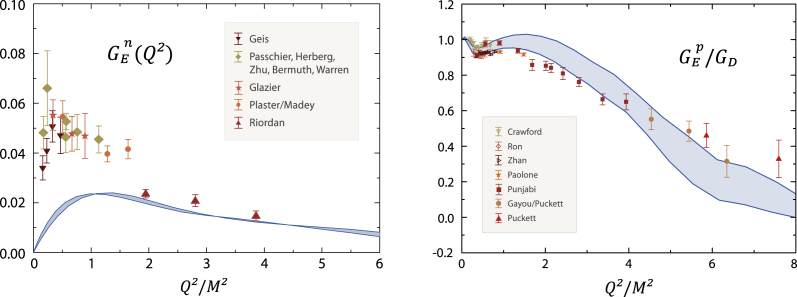
Electric form factors of the the neutron (*left panel*) and the proton normalized by the dipole (*right panel*), as functions of the photon momentum transfer and in comparison with experimental data. Figure adapted from Ref. [5].

Results for the pion-mass dependence and $Q^{2}$-evolution of various nucleon electromagnetic form factors are shown in Fig. 2, Fig. 3. The bands correspond to a variation of the infrared properties in the quark–gluon interaction. As anticipated, the absence of pion-cloud contributions in the chiral and low-momentum region is recovered in the results. All form factors are in reasonable agreement with experimental data at larger momentum transfer where the nucleon is probed at small length scales and the pion cloud becomes irrelevant. The missing structure mainly appears in the low-momentum region $Q^{2}≲2\;{\mathrm{GeV}}^{2}$. The calculated charge radii, such as the isovector Dirac radius in the left panel of Fig. 2, underestimate their experimental values but converge with lattice data at larger quark masses. Pion loops would increase the charge radii toward the chiral limit where they would diverge. Chiral effective field theory predicts that leading-order chiral corrections to proton and neutron anomalous magnetic moments carry an opposite sign; their magnitude is therefore enhanced in the isovector combination $\kappa^{v}=\kappa^{p}-\kappa^{n}$ and cancels in the isoscalar case $\kappa^{s}=\kappa^{p}+\kappa^{n}$. The isoscalar magnetic moment is quite accurately reproduced by the Faddeev calculation: $\kappa^{s}=-0.12\left(1\right)$, compared to the experimental value $\kappa_{\exp}^{s}=-0.12$ [5]. The calculated values of $\kappa^{s}$ and $\kappa^{v}$ correspond to an underestimation of 20%–30% in the proton and neutron magnetic moments $G_{M}^{p,n}\left(0\right)$. Another example is the neutron electric form factor $G_{E}^{n}\left(Q^{2}\right)$ in Fig. 3 which agrees with recent measurements at larger $Q^{2}$ but misses the characteristic bump at low $Q^{2}$. These observations suggest to identify the rainbow-ladder truncated nucleon with the ‘quark core’ in chiral effective field theories.

The large-$Q^{2}$ behavior of form factors is of great theoretical and experimental interest as well. The experimental falloff of the proton’s form factor ratio $G_{E}^{p}/G_{M}^{p}$ has been attributed to orbital angular-momentum correlations in the nucleon wave function which modify the perturbative scaling behavior and entail a zero crossing in $G_{E}^{p}\left(Q^{2}\right)$. Quark orbital angular momentum in terms of s, p and d waves appears in the Dirac–Lorentz structure of the nucleon’s rest-frame Faddeev amplitude. While nucleon and Δ baryons are dominated by s waves, p waves play an important role as well: they contribute ∼30% to the nucleon’s canonical normalization and diminish only slowly with increasing current–quark masses. The contribution from d waves, on the other hand, is below 1%. At large $Q^{2}$, the form-factor results from the Faddeev calculation become sensitive to the numerics; nevertheless, a decrease of $G_{E}^{p}$ compared to the dipole form is visible in Fig. 3 and implies a zero crossing as well.

Another remark concerns the timelike behavior of the form factors and the vector-meson dominance property which is a direct consequence of the underlying dynamics. The electromagnetic current is microscopically represented by the quark–photon vertex which can be separated in two terms: a Ball–Chiu part that satisfies electromagnetic gauge invariance, and another purely transverse term that includes vector-meson poles in the $J^{PC}=1^{--}$ channel [8], [9]. Since the rainbow-ladder truncation does not dynamically develop hadronic decay widths, the poles that are generated in the self-consistent calculation of the quark–photon vertex are timelike and real. The decomposition into ‘Ball–Chiu’ and ‘ρ-meson’ contributions can be made in all electromagnetic hadron form factors which therefore possess poles at $Q^{2}=-m_{\rho }^{2}$ and further $1^{--}$ excited-state locations. The transverse term is negative at spacelike $Q^{2}$ and, in the case of electric form factors, vanishes at $Q^{2}=0$, i.e., the Ball–Chiu part alone satisfies charge conservation $G_{E}^{p}\left(0\right)=1$. The ρ-meson term contributes roughly ∼50% to the nucleon’s squared charge radii throughout the current-mass range but has only a minor impact on its magnetic moments whose overall contribution comes from the Ball–Chiu term.

We note that a reduction of the Faddeev equation to a quark–diquark description, where scalar and axialvector diquark correlations are calculated from the same quark–gluon input, yields quite similar results for the form factors. The model dependence is however larger, especially at large $Q^{2}$, and the corresponding bands in Fig. 3 become sizeable; see also Fig. 5 below. Nevertheless, these results imply that the interaction of quarks with scalar and axialvector diquarks provides an overwhelming contribution to the nucleon’s binding.

## 3 Nucleon axial form factors

The Dyson–Schwinger/Faddeev approach was recently also applied to compute the nucleon’s axial and pseudoscalar form factors [6]. The respective current matrix elements are specified by the axial form factor $G_{A}\left(Q^{2}\right)$, the induced pseudoscalar form factor $G_{P}\left(Q^{2}\right)$, and the pseudoscalar form factor $G_{5}\left(Q^{2}\right)$:

(2) $$J_{5}^{\mu }=\Lambda_{+}\left(P_{f}\right)\gamma_{5}\left(G_{A}\left(Q^{2}\right)\;\gamma^{\mu }+G_{P}\left(Q^{2}\right)\;\frac{i\;Q^{\mu }}{2M_{N}}\right)\Lambda_{+}\left(P_{i}\right)\text{,}\;J_{5}=G_{5}\left(Q^{2}\right)\;\Lambda_{+}\left(P_{f}\right)\;i\gamma_{5}\;\Lambda_{+}\left(P_{i}\right)\text{.}$$

Their microscopic decomposition in the Faddeev framework is identical to Fig. 1 except for the type of $q\overset{¯}{q}$ vertices that are involved: the structure $\gamma^{\mu }$ that enters the self-consistent calculation of the quark–photon vertex is replaced by $\gamma_{5}\gamma^{\mu }$ and $\gamma_{5}$, respectively. Again, the pole structure of the resulting axial and pseudoscalar vertices allows us to extract information on the timelike behavior and identify the relevant scales in the form factors. $G_{A}$ is dominated by the $1^{++}$ axialvector meson $a_{1}\left(1260\right)$ and its excitations whereas $G_{P}$ and $G_{5}$ are governed by the pion pole. The pion–nucleon form factor $G_{\pi NN}$ is the residue of $G_{5}$ at the pion pole and thus related to the π(1300) and further $0^{-+}$ excitations. The Goldberger–Treiman relation $G_{A}\left(0\right)=f_{\pi }\;G_{\pi NN}\left(0\right)/M_{N}$ follows as a consequence of the axialvector Ward–Takahashi identity and analyticity which are satisfied microscopically.

**Fig. 4:**
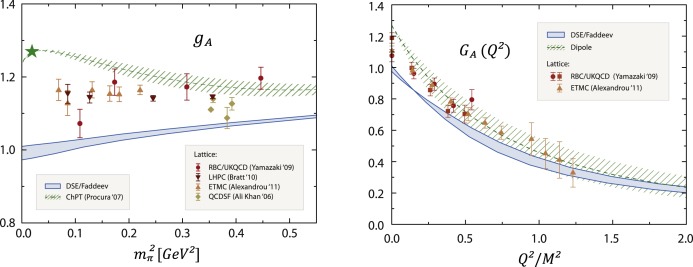
*Left panel:* Quark-mass dependence of the nucleon’s axial charge $g_{A}$, compared to lattice results and the chiral expansion of Ref. [10]. *Right panel:*$Q^{2}$-evolution of the axial form factor $G_{A}\left(Q^{2}\right)$, compared to lattice data and the experimental dipole form. Figure adapted from Ref. [6].

The (isovector) axial and pseudoscalar form factor results exhibit various similarities with their electromagnetic counterparts, see Fig. 4. The axial charge $g_{A}=G_{A}\left(0\right)$ underestimates the experimental value by 20%–25%; it falls below recent lattice data in the low quark-mass region and approaches the chiral expansion at larger pion masses. On the other hand, $G_{A}\left(Q^{2}\right)$ is consistent with the phenomenological dipole form at larger $Q^{2}$. Analogous results are obtained for the remaining pseudoscalar form factors. Once again, these features might be signals of missing pion-cloud effects. Such an interpretation was also suggested to explain the volume dependence of the lattice results for $g_{A}$ [11].

**Fig. 5:**
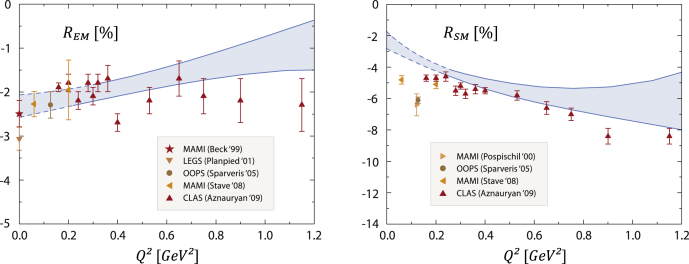
$Q^{2}$-dependence of the electric and Coulomb quadrupole form-factor ratios $R_{EM}$ and $R_{SM}$ compared to experimental data. Figure adapted from Ref. [7].

## 4 Δ(1232) and N→Δ transition form factors

Finally, the decomposition of Fig. 1 can be applied for the calculation of Δ(1232) and N→Δ transition form factors as well. Since a solution for the Δ bound-state amplitude from the Faddeev equation has become available only recently [12], we will restrict our discussion to the quark–diquark model. The derivation that leads to the diagrams in Fig. 1 yields analogous expressions in the quark–diquark approach [13], where the diquark ingredients can be computed self-consistently from the same quark–gluon input. Form-factor results exist for the Δ electromagnetic form factors [14], the ΔNπ pseudoscalar transition [15] as well as the NΔγ transition [7]. Experimental information on the Δ electromagnetic form factors is sparse; however, the quark–diquark results are in agreement with lattice data and reproduce the experimental value for the $\Omega^{-}$ magnetic moment. The pseudoscalar Δ→Nπ transition form factor is compatible with lattice data as well and also close to the experimental value. In the following we highlight recent results for the NΔγ transition; details can be found in Ref. [7].

The NΔγ transition has been accurately measured over a wide momentum range [16], [17]. It is dominated by a magnetic dipole transition which, in a quark-model picture, amounts to the spinflip of a quark and is related to the form factor $G_{M}^{⋆}\left(Q^{2}\right)$. The remaining electric and Coulomb quadrupole form factors are much smaller and expressed by the ratios $R_{EM}\left(Q^{2}\right)$ and $R_{SM}\left(Q^{2}\right)$ which encode the deformation in the transition. The analysis of pion electroproduction data via dynamical reaction models suggests that these ratios are almost entirely dominated by the pion cloud. In contrast, the quark–diquark results which are plotted in Fig. 5 reproduce the experimental data for $R_{EM}$ and $R_{SM}$ quite well, even without the inclusion of pion-cloud corrections. In the case of $R_{EM}$, this behavior originates from p-wave contributions in the nucleon and Δ bound-state amplitudes which are a consequence of Poincaré covariance. The removal of p waves results in a ratio that is overall positive and grows with increasing $Q^{2}$, with a trend towards the perturbative prediction $R_{EM}\to 1$ for $Q^{2}\to \infty$ [17]. The impact of d waves is almost negligible.

On the other hand, the result for the magnetic dipole transition form factor $G_{M}^{⋆}\left(Q^{2}\right)$ follows the characteristics of $G_{A}\left(Q^{2}\right)$ in Fig. 4: it agrees with experimental data at larger $Q^{2}$ and underestimates them by ∼25% at $Q^{2}=0$. This is consistent with the quark-model result and the expected behavior of the pion cloud from coupled-channel analyses. Moreover, neither $G_{M}^{⋆}$ nor $R_{SM}$ are sensitive to the addition of p and d waves but dominated by s-wave elements alone.

## 5 Conclusions and outlook

We have discussed several recent nucleon and Δ form factor results in the Dyson–Schwinger approach, either obtained directly via the covariant Faddeev equation or in a quark–diquark simplification. All calculations share the same quark–gluon input and the results display consistent features. Quark–quark correlations, which are mediated by a rainbow-ladder gluon-exchange interaction, can account for the overall properties of the nucleon and Δ quark core and justify a quark–diquark picture for these baryons. Dynamical chiral symmetry breaking and Poincaré covariance have important consequences for the behavior of form factors. Their timelike structure is dominated by meson poles in the underlying quark–antiquark vertices. The admixture of quark orbital angular momentum via p waves in s-wave dominated ground states is crucial for the NΔγ electric quadrupole form factor and the large-$Q^{2}$ behavior of electromagnetic form factors. The main missing ingredients in a rainbow-ladder approach are pion-cloud contributions at low momenta and small pion masses.

The combination of Dyson–Schwinger and covariant bound-state equations provides valuable tools for investigating the internal structure of hadrons. Its applications are still at an early stage, and it is imperative to extend the framework to study more sophisticated systems and processes. For example, an investigation of baryon excitations and nucleon-to-resonance transition form factors is desirable. The form-factor decomposition in Fig. 1 can also be generalized to compute a variety of hadron–photon and hadron–meson reactions such as virtual Compton scattering, pion electroproduction, pion–nucleon scattering, or timelike $p\overset{¯}{p}$ annihilation processes [18]. At the same time, these efforts must be complemented by technical improvements, such as residue calculus to provide kinematic access to truly large $Q^{2}$, or the implementation of pion-cloud corrections and hadronic decay channels via truncations beyond rainbow-ladder.
